# Comparison of two analyzer measurements focusing on material stiffness among normal, treatment-naïve, and treated glaucoma eyes

**DOI:** 10.1038/s41598-022-27346-w

**Published:** 2023-01-03

**Authors:** Shuichiro Aoki, Ryo Asaoka, Yuri Fujino, Shunsuke Nakakura, Hiroshi Murata, Yoshiaki Kiuchi

**Affiliations:** 1 Department of Ophthalmology, The University of Tokyo Graduate School of Medicine, Tokyo, Japan; 2 Department of Ophthalmology, Seirei Hamamatsu General Hospital, Hamamatsu City, Shizuoka Japan; 3 Seirei Christopher University, Hamamatsu City, Shizuoka Japan; 4 The Graduate School for the Creation of New Photonics Industries, Hamamatsu City, Shizuoka, Japan; 5 Department of Ophthalmology, Faculty of Medicine, Shimane University, Matsue, Japan; 6 Department of Ophthalmology, Saneikai Tsukazaki Hospital, Hyogo, Japan; 7 Department of Ophthalmology, National Center for Global Health and Medicine, Tokyo, Japan; 8 Department of Ophthalmology and Visual Science, Hiroshima University, Hiroshima, Japan

**Keywords:** Optic nerve diseases, Glaucoma

## Abstract

To investigate differences in biomechanical properties focusing on stiffness parameters between normal, treatment-naïve primary open-angle glaucoma (POAG), and treated POAG eyes. Retrospective case–control study, This study included 46 treatment-naïve POAG eyes, 46 POAG eyes treated with prostaglandin analogues, and 49 normal eyes used as controls; matched in terms of age and axial length. Corneal hysteresis (CH) and corneal resistance factor (CRF) were measured using an ocular response analyzer (ORA). Fifteen biomechanical parameters were measured with the Corneal Visualization Scheimpflug Technology (Corvis ST), including biomechanical glaucoma factor (BGF) and two stiffness parameters of ‘SP A1’ and ‘stress–strain index (SSI)’, which were compared among the three groups. Additionally, the area under the curve (AUC) values of the receiver-operating curve to discriminate control and treatment-naïve POAG eyes were calculated for BGF and CH. Treatment-naïve POAG eyes had higher ‘SSI’ than normal eyes even after controlling for IOP (*p* < 0.05, Tukey-Cramer test). Treated POAG eyes had significantly lower CRF, and higher BGF than treatment-naïve POAG eyes. There were also significant differences in CH or SP A1 among the three groups. BGF and CH had similar AUC values (0.61 and 0.59). Treatment-naïve POAG eyes had stiffer corneas compared to normal eyes, which seemed to result from the material/structure of the cornea rather than higher intraocular pressure. Antiglaucoma topical medication alters biomechanical properties measured with Corvis ST. These results are important for understanding the pathogenesis and improving the management of POAG.

## Introduction

Glaucoma is a leading cause of blindness worldwide. Elevated intraocular pressure (IOP) is crucial for the disease, because it is the main reason for the development of glaucoma; moreover, there is no other modifiable factor established for primary open-angle glaucoma (POAG)^1–5^. However, as has been revealed in previous studies, it cannot be the only factor in the development and progression of glaucoma, because IOP-lowering therapy cannot completely halt the progression of its irreversible visual field loss^6,7^. Thus, it is important to investigate the effect of variables other than elevated IOP on the development and progression of glaucoma.

The biomechanical properties of the eye play a significant role in the development and progression of glaucoma. In the last few decades, two instruments have become commercially available to quantify corneal biomechanical properties in clinics: the Ocular Response Analyzer (ORA; Reichert Inc., Depew, NY), and the Corneal Visualization Scheimpflug Technology (Corvis ST; Oculus GmbH, Wetzlar, Germany). The ORA provides corneal hysteresis (CH), which is derived from the viscoelastic property of the cornea^8^. In which, an air jet is applied to an eye, and CH is measured as the difference of the air-jet pressures at the events of the first (inward) and the second (outward) applanations. Low CH is associated with the diagnosis^9^, development^10^, severity^11^, and progression of glaucoma^12,13^. Whereas, in the Corvis ST, detailed images of the corneal deformation induced by the application of an air jet can be captured using an ultra-high-speed Scheimpflug camera. Previous studies have also confirmed the association between POAG severity^11^/VF progression^14^ of POAG and the Corvis ST parameters.

This study was conducted to elucidate two aspects of the biomechanical properties of glaucoma. First, Corvis ST has recently launched a novel glaucoma parameter of the Biomechanical Glaucoma Factor (BGF) for the purpose of discriminating normal tension glaucoma and normal eyes, based on 5 Corvis ST parameters^15^. We subsequently investigated the usefulness of this parameter, which resulted in the area under the curve (AUC) of 61% to diagnose glaucoma under medical treatments^16^. However, it is essential to analyze this aspect in treatment-naïve glaucoma eyes, i.e., excluding the influence of altered biomechanical properties due to the treatment such as topical prostaglandin analogues^17–26^. It was also our purpose to investigate various ORA and Corvis ST parameters comprehensively in glaucomatous eyes with and without treatments.

Second, recent studies have revealed the influence of stiff cornea (measured with Corvis ST) on the development and progression of glaucoma^16,27,28^. In particular, a recent study has suggested that the Corvis ST parameter of ‘stiffness parameter applanation 1 (SP-A1)’ (a high value is suggestive of stiff cornea) is a useful predictive parameter for the progression of glaucoma^27^. In SP-A1 calculation, the stiffness is quantified as a secant elastic modulus, that is, the ratio of the magnitude of applied stress to that of strain. SP-A1 is largely influenced by the IOP of an eye because higher IOP itself stiffens the cornea due to its nonlinear stress–strain relationship^8^. This may raise the question of whether the effect of a stiff cornea is due to the stiffness of the cornea purely as a material/structure, or that in conjunction with high IOP (probably regardless of treatment). The same is true for the corneal resistance factor (CRF), a stiffness parameter measured using ORA. In contrast to SP-A1 and CRF, the pure material stiffness of cornea can now be estimated using a newer Corvis ST parameter of the ‘Stress–Strain Index (SSI)’; it parametrizes the nonlinear relationship between stress and strain basing on finite-element models of an eye globe, and hence the bias of IOP can be avoided^29^. To this end, we compared the biomechanical properties measured with ORA and Corvis ST, including CH, CRF, BGF, SP-A1 and SSI, among 3 groups of normal controls, treatment-naïve POAG eyes, and treated POAG eyes. Our analysis confirmed significant and clinically relevant differences in several Corvis ST parameters; the cornea was stiffer even when the influence of high IOP was excluded in treatment-naïve POAG eyes than in control eyes. In addition, there were significant differences in the biomechanical properties between treatment-naïve and POAG eyes treated with prostaglandin analogues.

## Methods

This retrospective case–control study was designed to investigate the differences in biomechanical properties between normal, treatment-naïve POAG, and treated POAG eyes. This study was approved by the Research Ethics Committees of the Graduate School of Medicine and Faculty of Medicine at the University of Tokyo and Seirei Hamamatsu General Hospital (#10,619). This study was conducted in accordance with the tenets of the Declaration of Helsinki. All participants signed a written informed consent form for their clinical information to be stored in the hospital database and used for research.

### Participants

Primary open-angle glaucoma was diagnosed according to the following criteria: (1) typical glaucomatous changes in the optic nerve head (e.g., rim notch with a rim width ≤ 0.1 disc diameters, a vertical cup-to-disc ratio > 0.7, or a retinal nerve fiber layer defect); (2) glaucomatous VF defects compatible with the optic nerve head changes meeting the Anderson–Patella criteria^30^ on two consecutive examinations; (3) wide open angle with gonioscopy; and (3) no systemic or ocular history or existing factors that can cause secondary glaucomatous changes or elevation of IOP.

The treatment-naïve POAG group included patients with POAG who had not been prescribed any topical or systemic IOP-lowering medications before the time of measurements. The inclusion criteria of the treated group included patients with POAG who were treated with prostaglandin analogues for more than six months in either eye; use of other topical medications was allowed. If both eyes were eligible, one of the eyes was randomly selected for analysis.

Participants in the control group consisted of those who had undergone ophthalmologic examinations without any abnormal findings, except for clinically insignificant cataracts. If both eyes were eligible, one of the eyes was randomly selected for analysis.

The exclusion criteria for the three groups (control, treatment-naïve POAG, and treated POAG) were: age under 40 years; axial length > 28 mm; wearing contact lenses; any abnormality of the cornea that affects Corvis ST measurement such as corneal ectasia; and experience of any ophthalmological surgical/laser intervention, including cataract surgery, trabeculotomy, trabeculectomy, laser trabeculoplasty, corneal refractive surgery, or scleral buckling. The three groups of control, treatment-naïve POAG, and treated POAG were matched to each other for age and axial length, which may have nonnegligible effects on the biomechanical properties of the eye^31,32^.

Axial length was measured using IOL Master versus 5.02 (Carl Zeiss Meditec, CA, USA). The visual field was measured using the Humphrey Field Analyzer II (Carl Zeiss Meditec Inc., Dublin, CA, USA) with a 24–2 or 30–2 SITA-standard program. The axial length and visual field were measured within 3 months of the Corvis ST and ORA measurements. The Goldmann applanation tonometer (GAT)-IOP was also measured on the same day as the Corvis ST and ORA measurements. Corvis ST and ORA measurements were conducted at 15 min intervals. The order of the measurements was randomly determined.

### Corvis ST

The principles of Corvis ST measurements have been described in detail elsewhere^33^. The high-speed Scheimpflug camera recorded 140 images of the cornea before and after a transient indentation of the cornea, which occurred within 30 ms after the application of an air impulse. Corneal response is characterized by two applanations during inward and outward corneal movements, which occur before and after the maximum displacement of the corneal apex. Because the cornea is viscoelastic, it dissipates some of the applied energy, and the corneal shape at the second applanation during the outward movement of the cornea is different from that at the first applanation. The parameters examined in this study included central corneal thickness (CCT) and various corneal morphological findings at the time of the two applanations and the maximum displacement of the corneal apex, as detailed in Table 1. All parameters were calculated using the current version of the Corvis ST software (version 1.6r2223). The precise descriptions of the representative Corvis ST parameters are as follows: ‘biomechanical IOP (bIOP)’ is the estimate of IOP adjusted for CCT and age using the finite element method^34^; ‘Integrated inverse radius’ is the integration of curvature during the concave state of the cornea; and ‘deformation amplitude (DA) ratio 1 mm’ is the ratio between the deformation amplitude of the apex to that at the points located 1 mm on either side of the apex. The steep indentation of the cornea can be represented by a high integrated inverse radius or a high DA ratio 1 mm, suggesting a soft cornea.

**Table 1:** Summary of Corvis-ST measured parameters. DA: deformation amplitude, HC: highest concavity.

Name	Abbreviation	Description
A1 time (ms)	–	Time of the first applanation
A1 velocity (m/s)	–	Velocity of the corneal apex at the first applanation
A2 time (ms)	–	Time of the second applanation
A2 velocity (m/s)	–	Velocity of the corneal apex at the second applanation
HC time (ms)	–	Time of the highest concavity
HC deflection amplitude (mm)	–	Deflection amplitude of the HC
Peak distance (mm)	–	Distance between nondeformed peaks
Radius (mm)	–	Radius of curvature at maximum deformation
Integrated inverse radius (mm^–1^)	–	The integration of curvature during the concave state of the cornea
DA ratio 1 mm		The ratio between the deformation amplitude of the apex and the average of two points located 1 mm on either side of the apex
Whole eye movement max (mm)	–	Maximum displacement of eye globe due to air puff application
Pachyslope	–	Represents the difference in corneal thickness from the corneal apex toward the periphery
Biomechanical intraocular pressure (mmHg)	bIOP	Corrected estimate of IOP obtained adjusted for central corneal thickness and age
Stress–strain index	SSI	Represents material stiffness of the cornea
Biomechanical glaucoma factor	BGF	Comprised of five parameters to distinguish normal tension glaucoma

**Figure 1:**
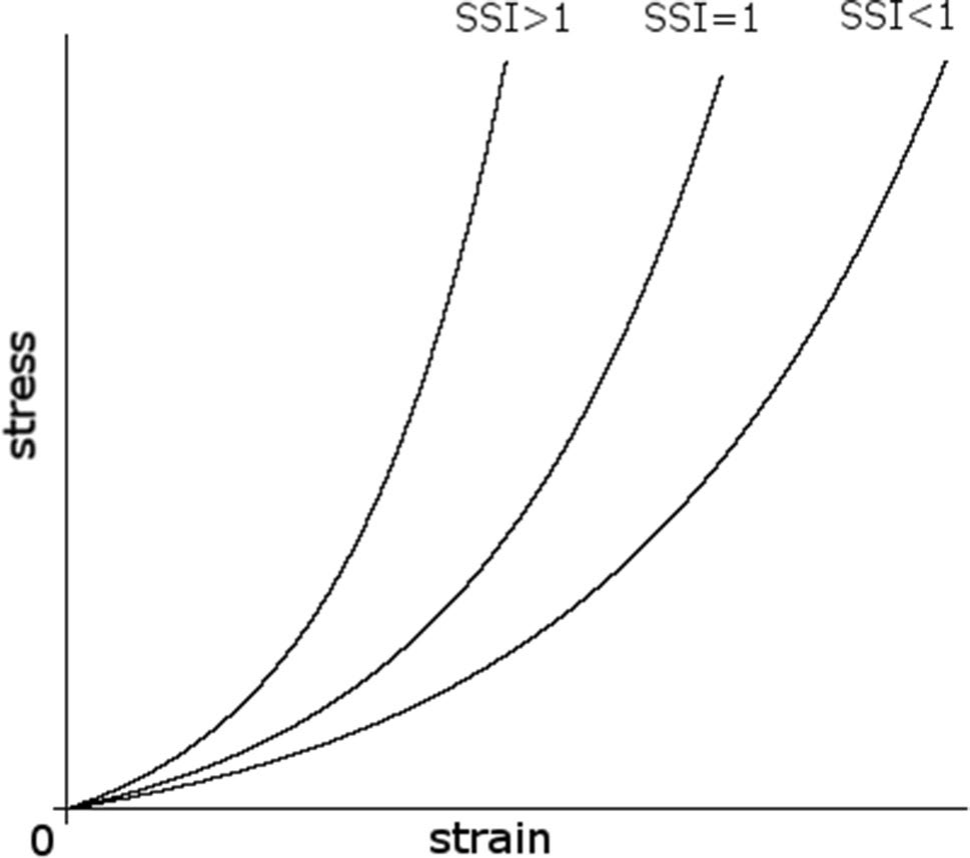
Stress–strain relationship and stress–strain index. Stress–strain curves for eyes with three different SSIs are illustrated. Higher SSI value indicates smaller strain (less deformation) under the same stress.

Corvis ST provides several stiffness parameters of cornea, out of which ‘SP A1’ and ‘SSI’ were the main interests in this study. SP A1 is the ratio of stress on the cornea over its displacement at the first applanation and represents the elastic modulus of the cornea:

$${\text{SP A}}1 = \frac{{{\text{adjAP}}1{ }{-}{\text{ bIOP}}}}{A1DeflAmp}$$

where adjAP1 represents the adjusted air pressure on the cornea at the first applanation and A1DeflAmp represents the deflection amplitude at the first applanation. Higher SP A1 value indicates a stiffer cornea. SP A1 assesses the secant elastic modulus at a single state of the cornea, measured as the ratio of stress over strain. SP A1 largely depends on IOP and corneal geometry (ex. CCT). This is because the nonlinear relationship between IOP and corneal morphology and the elastic properties of the cornea are not considered in the formula^8^. In contrast, SSI parameterizes this nonlinear profile of the stress–strain relationship of the eye (Fig. 1). More specifically, SSI was developed using finite element models of an eye globe at different levels of IOP so that it is independent of IOP and corneal geometry. Thus, the SSI represents the pure material stiffness of the cornea^29^. An SSI value of 1 indicates the average value of a normal eye in a 50-year-old individual. A higher SSI value indicates a less deformable and stiffer cornea.

Biomechanical glaucoma factor (BGF) is calculated using the formula below^15^;

$$Beta= 34.128+2.64*DARatioProg-0.641*HCTime-0.049*PachySlope-0.202*bIOP-0.036*CCT$$

$$BGF =\frac{EXP\left(Beta\right)}{EXP\left(Beta\right)+1}$$

BGF ranges from 0 to 1, where a high value indicate a high likelihood of glaucoma^15^. The 5 Corvis ST parameters ($RatioProg, HCTime, PachySlope,bIOP,\mathrm{ and }CCT$) comprising the BGF formula are detailed elsewhere^15,16^. Deformation amplitude ratio progression (DARatioProg) represents the increase ratio of the deformation amplitude from the corneal apex toward the periphery; higher DARatioProg value indicates a stiffer cornea. Pachyslope represents the change in corneal thickness from the corneal apex toward the periphery; a smaller Pachyslope value indicates a relatively thin cornea in the periphery compared with the central region.

Corvis ST measurements were conducted in triplicate. Only reliable Corvis ST measurements were used based on the “OK” quality index displayed on the device monitor. The average values were used in the analysis.

### ORA measurement

Details of the ORA measurement are summarized elsewhere^35^. Similarly to Corvis ST, in the ORA measurement, an air pulse deflects the cornea and the cornea experiences two applanation events. After a certain amount of time has elapsed from the moment of initial corneal applanation, the jet flow diminishes. Because the cornea is viscoelastic, some energy dissipates, so the air jet pressure at the second applanation (P2) during the outward movement of the cornea is lower than the pressure at the first applanation (P1). This pressure difference at the two applanations (P1–P2) is referred to as CH. Corneal hysteresis reflects the damping capacity or energy dissipation of the cornea^8,36,37^. Corneal resistance factor is an indicator of the overall “resistance” or the elastic properties of the cornea. CRF is derived as (P1 – k × P2), where constant k is empirically determined so that CRF is strongly associated with CCT^36,37^. Corneal resistance factor, as well as SP A1 and unlike SSI, depends on IOP^38^. The ORA measurement was measured in triplicate. Only measurements with a quality index of > 6.5 were used, and the average value were used in the analysis.

### Statistical analysis

Age, axial length, and Corvis ST and ORA parameters were compared between the control, treatment-naïve POAG, and treated POAG groups using the Tukey–Kramer test. The differences in Corvis ST and ORA parameters were further evaluated, using multivariate linear regression model fit with group (control, treatment-naïve, and treated) and bIOP for each parameter. The model was controlled for bIOP because treatment-naïve group showed a significantly larger GAT-IOP and bIOP than other groups, which potentially affects biomechanical parameters. Furthermore, AUC values of the receiver-operating curve to discriminate control and treatment-naïve POAG eyes, as well as control and treated POAG eyes, were calculated and compared between CH and BGF using DeLong test. All data processing and analyses were performed using the statistical programming language, R (The R Foundation for Statistical Computing, Vienna, Austria).

## Results

We enrolled 49, 46, and 46 eyes in the control, treatment-naïve POAG, and treated POAG groups, respectively. The participants’ basic characteristics are listed in Table 2. There were no significant differences in age, axial length, or CCT among the three groups. GAT-IOP and bIOP were significantly higher in the treatment-naïve group than in the other two groups (*p* < 0.05, Tukey–Kramer test). Mean ± standard deviation [range] of the mean deviation value of HFA 24–2/30–2 test for treatment-naïve POAG group was − 5.81 ± 6.73 [ − 27.79, 2.15] dB.

**Table 2:** Basic characteristics of subjects.

Characteristics	Control (n = 49)	treatment-naive POAG (n = 46)	treated POAG (n = 46)	*p* value (control vs. treatment-naïve)	*p* value (control vs. treated)	*p* value (treatment-naive vs. treated)
Age (years)	71.9 ± 13.6 [ 40–90 ]	68.9 ± 11.9 [ 44–92 ]	70.1 ± 9.9 [ 43–92 ]	0.44	0.75	0.88
Axial length (mm)	23.91 ± 1.40 [ 21.4–27.6 ]	24.29 ± 1.57 [ 22.0–27.6 ]	24.39 ± 1.19 [ 22.2–27.4 ]	0.38	0.21	0.94
GAT-IOP (mmHg)	13.6 ± 2.9 [ 8–19 ]	16.2 ± 3.3 [ 11–25 ]	13.7 ± 2.6 [ 8–20 ]	< 0.0001	0.95	0.00036
bIOP (mmHg)	12.55 ± 2.59 [ 7.7–20.2 ]	13.70 ± 2.20 [ 8.7–19.4 ]	12.31 ± 1.63 [ 7.9–15.7 ]	0.031	0.85	0.0077
CCT (μm)	533.8 ± 33.9 [ 455–618 ]	537.4 ± 40.3 [ 422–632 ]	525.5 ± 37.9 [ 455–623 ]	0.88	0.53	0.28

*GAT-IOP* Goldmann applanation tonometry measured intraocular pressure, *bIOP* biomechanical IOP, *CCT* central corneal thickness.

A comparison of biomechanical parameters measured using the ORA and Corvis ST is summarized in Table 3. CH was significantly lower in the treated POAG group than that in the control group (*p* = 0.0011); CRF was again significantly lower in the treated POAG group than those in the other two groups (*p* = 0.0022 and < 0.0001). Compared to the control group, the treatment-naïve POAG group had a higher SSI (*p* < 0.05, Tukey–Kramer test), suggesting stiffer corneas in the treatment-naïve POAG group. The difference was still significant after controlling for bIOP, CCT, and axial length in the multivariate linear regression model, as shown in Table 4. Furthermore, compared to the control group, the treatment-naïve POAG group had a significantly shorter A2 time, smaller magnitude of A2 velocity, lower peak distance and HC deflection amplitude (*p* < 0.05, Table 4). There were no significant differences in CH, BGF, or SPA1 levels between the control and treatment-naïve POAG group.

**Table 3:** comparisons of biomechanical parameters measured with ORA and Corvis ST.

Parameter	Control	Treatment-naive POAG	treated POAG	*p* value (control vs. treatment-naïve)	*p* value (control vs. treated)	*p* value (treatment-naive vs. treated)
CH (mmHg)	10.02 ± 1.10 [ 7.51–12.06 ]	9.71 ± 1.13 [ 6.35–12.48 ]	9.18 ± 1.11 [ 7.08–11.72 ]	0.36	0.0011	0.066
CRF (mmHg)	9.41 ± 1.57 [ 5.89–13.43 ]	9.87 ± 1.20 [ 7.10–12.44 ]	8.38 ± 1.57 [ 5.93–12.95 ]	0.28	0.0022	< 0.0001
A1 Time (ms)	7.07 ± 0.33 [ 6.44–8.23 ]	7.28 ± 0.32 [ 6.78–8.13 ]	7.19 ± 0.22 [ 6.76–7.66 ]	0.0027	0.12	0.37
A1 velocity (m/s)	0.16 ± 0.02 [ 0.10–0.18 ]	0.14 ± 0.02 [ 0.09–0.18 ]	0.16 ± 0.02 [ 0.11–0.19 ]	0.0066	0.81	0.0011
A2 time (ms)	22.22 ± 0.44 [ 21.02–23.23 ]	21.87 ± 0.48 [ 20.18–22.86 ]	21.92 ± 0.39 [ 21.12–22.78 ]	0.00048	0.0033	0.84
A2 velocity (m/s)	− 0.29 ± 0.03 [ − 0.37–− 0.22 ]	− 0.26 ± 0.03 [ − 0.30–− 0.18 ]	− 0.28 ± 0.03 [ − 0.37–− 0.22 ]	< 0.0001	0.011	0.032
HC time (ms)	17.02 ± 0.52 [ 15.55–18.13 ]	16.98 ± 0.40 [ 15.94–17.71 ]	17.22 ± 0.46 [ 16.00–18.14 ]	0.93	0.083	0.038
HC deflection Amplitude (mm)	0.99 ± 0.11 [ 0.68–1.22 ]	0.91 ± 0.10 [ 0.57–1.06 ]	0.95 ± 0.11 [ 0.75–1.18 ]	0.0031	0.25	0.2
Peak distance (mm)	5.14 ± 0.30 [ 4.31–5.80 ]	4.94 ± 0.29 [ 4.03–5.37 ]	5.07 ± 0.24 [ 4.58–5.51 ]	0.0016	0.39	0.078
Radius (mm)	7.15 ± 0.66 [ 5.62–8.49 ]	7.47 ± 0.76 [ 5.62–9.33 ]	7.40 ± 0.68 [ 5.96–9.15 ]	0.07	0.2	0.88
Integrated inverse radius (mm^−1^)	8.69 ± 1.02 [ 6.85–11.00 ]	8.13 ± 1.10 [ 5.00–10.64 ]	8.39 ± 0.95 [ 6.29–10.29 ]	0.024	0.33	0.45
DA ratio 1 mm	1.59 ± 0.05 [ 1.51–1.71 ]	1.59 ± 0.05 [ 1.47–1.71 ]	1.62 ± 0.05 [ 1.52–1.74 ]	0.93	0.021	0.0086
Whole eye movement max (mm)	0.37 ± 0.09 [ 0.14–0.65 ]	0.35 ± 0.07 [ 0.20–0.53 ]	0.32 ± 0.06 [ 0.18–0.45 ]	0.46	0.0046	0.12
Pachyslope	40.25 ± 11.66 [ 10.16–78.32 ]	40.69 ± 12.03 [ 11.74–63.99 ]	36.08 ± 9.78 [ 13.87–60.68 ]	0.98	0.17	0.12
BGF	0.58 ± 0.24 [ 0.06–0.95 ]	0.49 ± 0.24 [ 0.05–0.91 ]	0.61 ± 0.24 [ 0.09–0.95 ]	0.13	0.83	0.035
SP A1	96.54 ± 20.37 [ 55.53–147.18 ]	101.29 ± 19.11 [ 63.11–158.59 ]	97.71 ± 16.34 [ 71.81–137.53 ]	0.43	0.95	0.63
SSI	1.17 ± 0.22 [ 0.71–1.79 ]	1.31 ± 0.29 [ 0.87–2.43 ]	1.29 ± 0.27 [ 0.85–2.07 ]	0.028	0.077	0.91

*BGF* biomechanical glaucoma factor; *CH* corneal hysteresis; *CRF* corneal resistance factor; *DA* deformation amplitude; *HC* highest concavity; *SP A1* stiffness parameter applanation 1; *SSI* stress–strain index.

**Table 4:** Comparisons of biomechanical parameters measured with ORA and Corvis ST.

Parameter	ANCOVA	Control versus treatment-naïve			Treatment-naïve versus treated			Control versus treated		
	*p* value	β	*p* value	R2	β	*p* value	R2	β	*p* value	R2
CH (mmHg)	0.00052	− 0.46	0.11	0.17	− 0.33	0.19	0.36	− 0.67	0.0022	0.39
CRF (mmHg)	< 0.0001	− 0.051	0.86	0.53	− 0.65	0.0078	0.72	− 0.68	0.0026	0.66
A1 time (ms)	< 0.0001	0.054	0.13	0.85	0.13	0.00033	0.8	0.18	< 0.0001	0.91
A1 velocity (m/s)	< 0.0001	− 0.0046	0.21	0.59	0.0013	0.7	0.62	− 0.0015	0.49	0.61
A2 time (ms)	< 0.0001	− 0.18	0.017	0.66	− 0.22	0.01	0.52	− 0.38	< 0.0001	0.75
A2 velocity (m/s)	< 0.0001	0.028	< 0.0001	0.55	− 0.0025	0.7	0.35	0.025	< 0.0001	0.42
HC time (ms)	0.03	0.055	0.69	0.11	0.16	0.19	0.14	0.2	0.05	0.21
HC deflection Amplitude (mm)	< 0.0001	− 0.044	0.0024	0.78	− 0.031	0.043	0.72	− 0.072	< 0.0001	0.74
Peak distance (mm)	< 0.0001	− 0.13	0.0024	0.74	− 0.045	0.3	0.68	− 0.17	< 0.0001	0.73
Radius (mm)	0.049	0.32	0.067	0.24	0.22	0.19	0.32	0.36	0.0072	0.27
Integrated inverse radius (mm^− 1^)	0.0032	− 0.31	0.13	0.5	− 0.38	0.037	0.61	− 0.53	0.00095	0.55
DA ratio 1 mm	0.00032	0.0065	0.61	0.39	0.0063	0.58	0.51	0.017	0.014	0.65
Whole eye movement max (mm)	0.004	0.0025	0.88	0.24	− 0.042	0.021	0.18	− 0.044	0.0071	0.28
Pachyslope	0.097	− 1.8	0.61	0.11	− 2	0.52	0.15	− 4.1	0.079	0.11
BGF	0.00080	− 0.018	0.7	0.56	− 0.0034	0.92	0.64	− 0.021	0.49	0.71
SP A1	0.047	− 2.6	0.35	0.76	7.5	0.0078	0.71	6.3	< 0.0001	0.87
SSI	0.0071	0.14	0.011	0.38	0.097	0.12	0.4	0.19	< 0.0001	0.53

Differences among three groups were tested by analysis of covariance; Differences between two groups from three groups were tested with a linear multiple regression model adjusted for bIOP, central corneal thickness, and axial length.

*BGF* biomechanical glaucoma factor; *bIOP* biomechanical intraocular pressure; *CH* corneal hysteresis; *CRF* corneal resistance factor; *DA* deformation amplitude; *HC* highest concavity; *SP A1* stiffness parameter applanation 1; *SSI* stress–strain index.

Compared to the treatment-naïve POAG group, the treated POAG group had significantly lower CRF, higher magnitude of A1 and A2 velocity, larger HC time, higher DA ratio 1 mm, and higher BGF (*p* < 0.05, Tukey–Kramer test). After controlling for bIOP, CCT, and axial length in the multivariate linear regression model, the treated POAG group had significantly lower CRF, higher A1 time, lower A2 time, lower HC deflection amplitude, lower integrated inverse radius, and lower whole eye movement max [mm]. These differences suggest a stiffer cornea in the treated POAG group. There were no significant differences in SPA1 or SSI between the treatment-naïve POAG and treated POAG group.

**Figure 2:**
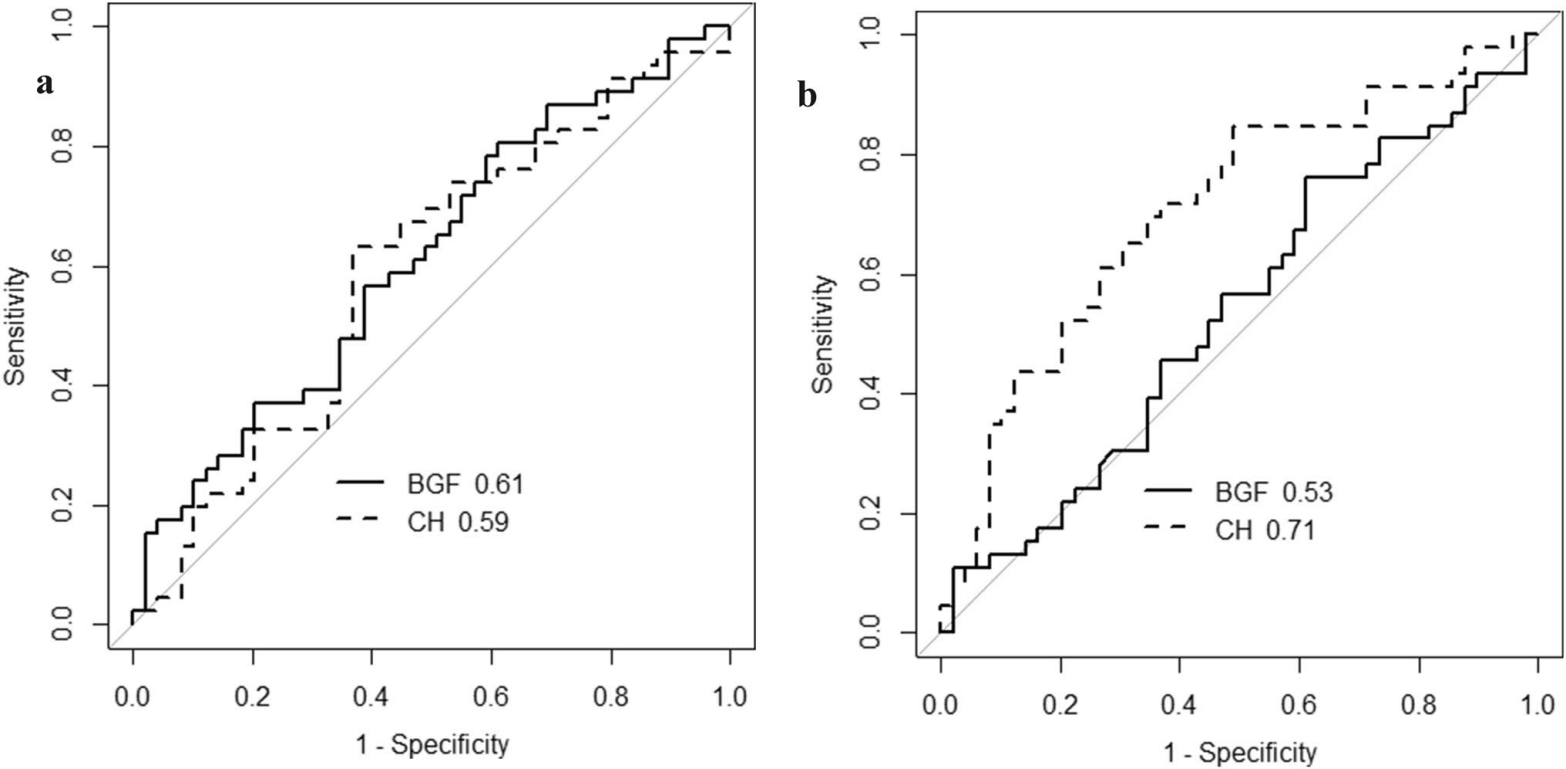
Receiver-operating curves to discriminate control and treatment-naïve POAG eyes (**a**) as well as control and treated POAG eyes (**b**) for CH and BGF. BGF: biomechanical glaucoma factor, CH: corneal hysteresis.

Figure 2A shows the receiver-operating characteristic curve to discriminate between control and treatment-naïve eyes with POAG for BGF and CH. The AUC values were 0.61 and 0.59 for BGF and CH, respectively. There was no significant difference between these values (*p* = 0.85, DeLong test). Figure 2B shows the curve to discriminate control and treated POAG eyes, with AUC values of 0.53 and 0.71 for BGF and CH, respectively. A significant difference was observed between these values (*p* = 0.0012, DeLong test).

## Discussion

In the current study, we compared biomechanical properties measured with ORA and Corvis ST among three groups: normal controls, treatment-naïve POAG eyes, and treated POAG eyes. As a result, several Corvis ST-related parameters were found to be significantly different among the three groups. In general, these differences suggest that the cornea was stiffer in the treatment-naïve POAG group than that in the control group. Biomechanical properties were altered in treated POAG eyes compared to treatment-naïve POAG eyes, where the cornea was softer in the former group. Our investigation of SP A1 and SSI revealed that these findings were not simply derived from the higher IOP in the treatment-naïve POAG eyes, but from the material/structure of the cornea itself in the group.

Treatment-naïve POAG eyes had higher SSI than control eyes, even after controlling for bIOP, while there was no significant difference in SP A1 among control, treatment-naïve, and treated POAG eyes. These results suggest that corneal material/structural stiffness is associated with the pathogenesis of POAG. Stiffer corneas imply stiffer lamina cribrosa and peripapillary sclera, potentially leading to greater optic nerve head vulnerability. Furthermore, stiffer ocular tissues may have lower energy dissipation or damping capacity. We have argued in previous studies^39,40^ that dynamic changes in IOP and eye globe deformation caused by daily phenomena such as pulsation^41^, blinking^42,43^, or eye movements^44,45^, may cause damage to the optic nerve head, and that eyes with smaller energy dissipation may be more vulnerable to such stress and prone to faster progression of glaucoma. Quassim et al. ^27^ recently reported that a higher SP A1 value was significantly associated with faster structural and functional progression of glaucoma. However, in their analysis, a correlation between baseline IOP (measured with GAT or Corvis ST) and baseline SP A1 was observed. Thus, a higher baseline IOP might have affected subsequent glaucoma progression regardless of treatment. Our results (higher SSI) suggested that the cornea in the treatment-naïve POAG eyes was stiffer, even if the influence of IOP was excluded, further supporting the contribution of biomechanical factors other than IOP to glaucoma pathogenesis.

Corneal resistance factor represents corneal stiffness, not excluding the effect of IOP, in principle, similar to SPA1, and unlike SSI. De Moraes et al. reported that in a multivariate analysis, CH, but not CRF, was significantly associated with the rate of visual field change in glaucoma population with various subtypes^13^. Another retrospective study found no significant relationship between CRF and the rate of progression speed in normal tension glaucoma^46^. In the present study, CRF was higher in treatment-naïve POAG than in other two groups. This is in contrast to SP A1, which showed no significant differences among the three groups. Considering the significantly lower IOP in the treated POAG group, these findings may suggest that CRF is more sensitive as a stiffness index (including the effect of IOP) than SP A1.

A limited number of studies have investigated corneal biomechanical properties in treatment-naïve POAG eyes compared to those in normal eyes. Recently, Miki et al. compared biomechanical parameters measured with Corvis ST between 35 normal healthy eyes and treatment-naïve normal tension glaucoma eyes^28^. They found higher HC deflection amplitude and peak distance, which suggests more deformable corneas in glaucomatous eyes, which contradicts our results. The exact reason for these different results is unclear, however they could be attributed to the population differences ; Miki et al. examined a population with longer average axial length of 25.8 mm compared with those in our study (23.91 mm and 24.29 mm for controls and treatment-naïve POAG eyes, respectively) since eyes with longer axial length exhibits greater compliance in Corvis ST measurement^32,47,48^. A relatively small number of the samples (35 eyes) may be another reason. Wu et al. compared Corvis ST parameters between 19 normal eyes, 35 treatment-naïve POAG eyes, and 34 POAG eyes treated with prostaglandin analogues^20^. They found that treatment-naïve POAG eyes had smaller HC deformation amplitude than normal eyes. This is consistent with our results; however, it was not possible to analyze SPA1 and SSI because of the older version of Corvis ST software. In the current study, we evaluated, SSI in normal eyes, treatment-naïve POAG eyes, and treated POAG eyes, for the first time. As a result, it was suggested that treatment-naïve POAG eyes had a stiffer cornea in the material/structure, independent of IOP level.

Biomechanical glaucoma factor is an index for discriminating normal-tension glaucoma from normal healthy eyes and consists of five Corvis ST parameters. Our previous study compared BGF between healthy eyes and treated POAG eyes and it was suggested that no significant difference between these two groups^16^. In accordance with this result, current results suggested no significant difference in BGF between normal and treatment-naïve POAG eyes. Moreover, the AUC values obtained were very similar between the two studies (both 0.61). In summary, topical antiglaucoma medication alters the biomechanical properties of the cornea^20,22^, However our findings indicate only a marginal effect on the usefulness of BGF when diagnosing glaucoma.

Previous studies indicated that CH measured with ORA is useful when diagnosing^9^, monitoring the development^10^, and conducting management^12,13^ of glaucoma. Many studies have reported CH in normative (9.77–10.20 mmHg)^49–51^ and also treated glaucomatous eyes (7.5–9.5 mmHg)^12–14,35^. The current study revealed comparative values (10.02 and 9.18 mmHg for control and treated POAG eyes, respectively). CH was significantly lower in the treated POAG group, as previously reported^9,50^. However, no significant difference was observed in CH between the control and treatment-naïve POAG groups, with the AUC value as low as 0.59. Few studies have investigated CH in treatment-naïve glaucomatous eyes, some of which were lower than that in the current treatment-naïve POAG eyes (9.71 mmHg)^52,53^. A prospective study by Susanna et al. indicated that CH is a risk factor for the development of glaucoma even when a multivariable model to adjust for the treatment was used^10^. Boliver et al.^52^ found that CH (8.9 mmHg in average) was associated with the amount of VF damage in treatment-naïve OAG eyes. Prata et al.^53^ found an association between CH (8.1 mmHg in average) and optic nerve head morphology in untreated newly diagnosed patients with POAG. These lower values could be attributed to the difference in IOP levels. These previous studies were the investigations in eyes with POAG mainly including eyes with IOP > 20 mmHg, whereas most of the eyes in the current study had normal tension glaucoma (GAT-IOP from 8 to 19 mmHg), reflecting its high prevalence in the region in which this study was conducted^54^. This difference would yield a nonnegligible effect on CH, because CH increases with the decrease in IOP^55^; lower IOP levels in the current study may have resulted in higher CH values in treatment-naïve POAG eyes. Indeed, Chen et al. reported no significant difference in CH between treatment-naïve normal tension glaucomatous eyes and normal eyes (9.1 vs. 8.9 mmHg), although it was suggested that CH is useful when screening for glaucoma^56^. In addition, Park et al.^57^ reported although there was a significant difference in CH among normal, early, and advanced treatment-naïve normal tension glaucoma eyes (10.83, 10.56, and 9.78 mmHg, respectively), however early treatment-naïve glaucoma eyes had CH value comparable to normal eyes. This may imply that the current study predominantly included eyes with early treatment-naïve glaucoma eyes, as suggested by the HFA visual field test mean deviation value of − 5.81 in average, and the CH value was not significantly different from that in normal eyes. The usefulness of CH in (early) treatment-naïve normal-tension glaucoma needs to be revisited in the future with a larger dataset with different disease severities. On the other hand, in our previous reports, the progression of glaucoma was faster with lower CH in mostly normal tension glaucoma eyes^16,39,40^. This may, together with the present finding that CH in treatment-naïve POAG eyes was comparable to that in normal eyes, imply that CH is not involved in the pathogenesis of normal tension glaucoma itself, but may reflect the change associated with the therapeutic interventions. To investigate this hypothesis, longitudinal observation of CH before and after the initiation of topical medication in normal-tension glaucoma and assessment of the contribution of CH to progression are needed.

Our multivariate analysis further showed that, compared to treatment-naïve POAG eyes, treated POAG eyes exhibited longer A1 time, shorter A2 time, smaller HC deflection amplitude, lower integrated inverse radius, and higher SP-A1, suggestive of stiffer cornea. Accumulating evidence indicates that the use of topical prostaglandin analogues can affect the structure and material properties of the cornea and anterior sclera^23–26^, possibly by upregulating matrix metalloproteinase. In particular, some reports have suggested that the cornea and anterior sclera became thinner with the initiation of prostaglandin analogues^24,25^, which is consistent with our results of an insignificantly lower CCT in treated POAG eyes than in treatment-naïve POAG eyes.

Furthermore, other biomechanical parameters may change due to prostaglandin analogues usage^17–22^. Previous reports have reported conflicting results regarding the effect of prostaglandin analogues on Corvis ST parameters in POAG eyes. In a prospective study, Wu et al. reported that POAG eyes became less deformable after commencing topical use of prostaglandin analogues^20^. In contrast, Yasukura et al. retrospectively examined changes in Corvis-ST parameters in POAG eyes and reported greater corneal compliance after prostaglandin analogues use^22^. It should be noted that the reduction of IOP with topical medication itself also alters the biomechanical response as measured by ORA and Corvis ST^8,55,58–60^, making the interpretation of the effect of prostaglandin analogues on corneal stiffness in a complex, because most of the treated POAG eyes used anti-glaucomatous eye drops other than prostaglandin analogues, as is often observed in real-world clinics. Softer cornea in the treated POAG eyes than in the treatment-naïve POAG eyes suggested in monovariate analysis may be at least partly explained by the lower IOP in the former group (12.31 vs. 13.70 mmHg in bIOP^58–60^, but a future study would be needed to investigate more in detail regarding the change of corneal stiffness with the initiation of prostaglandin analogues, using both SP A1 and SSI; corneal stiffness with and without the bias by IOP.

Our study had a few limitations. First, it included a relatively small number of patients. A larger dataset should be used to confirm the results of this study. Second, a longitudinal observation of the effect of the change in biomechanical properties caused by IOP-lowering medication on the progression of glaucoma is needed. Third, similar analyses should be performed to further confirm similar findings in different types of glaucoma, such as primary angle closure glaucoma and exfoliation glaucoma. We could not assess the effect of antiglaucoma medications other than prostaglandin analogs on corneal biomechanical parameters in treated POAG eyes because of heterogeneity of prescribed agents other than PG analogues. There are only limited number of studies investigated this issue; a previous study suggested no effect by carbonic anhydrase^61^. However, this issue should be investigated in a future study.

In conclusion, the Corvis ST parameters of BGF and other ORA parameters of CH had similar and low discrimination abilities between treatment-naïve early stage POAG (AUC = approximately 0.60) and normal eyes. Treatment-naïve POAG eyes have a stiffer cornea than normal eyes, and anti-glaucoma therapy has a significant impact on biomechanical properties measured with Corvis ST. These results could potentially help to understand the pathogenesis of POAG and better utilize assays of biomechanical properties in clinical settings.

## Data Availability

All data is available if requested to the corresponding author.
