# Author Correction: Comparison of two analyzer measurements focusing on material stiffness among normal, treatment‑naïve, and treated glaucoma eyes

**DOI:** 10.1038/s41598-024-56012-6

**Published:** 2024-03-05

**Authors:** Shuichiro Aoki, Ryo Asaoka, Yuri Fujino, Shunsuke Nakakura, Hiroshi Murata, Yoshiaki Kiuchi

**Affiliations:** 1 Department of Ophthalmology, The University of Tokyo Graduate School of Medicine, Tokyo, Japan; 2 Department of Ophthalmology, Seirei Hamamatsu General Hospital, Hamamatsu City, Shizuoka Japan; 3 Seirei Christopher University, Hamamatsu City, Shizuoka Japan; 4 The Graduate School for the Creation of New Photonics Industries, Hamamatsu City, Shizuoka Japan; 5 Department of Ophthalmology, Faculty of Medicine, Shimane University, Matsue, Japan; 6 Department of Ophthalmology, Saneikai Tsukazaki Hospital, Hyogo, Japan; 7 Department of Ophthalmology, National Center for Global Health and Medicine, Tokyo, Japan; 8 Department of Ophthalmology and Visual Science, Hiroshima University, Hiroshima, Japan

Correction to: *Scientific Reports* 10.1038/s41598-022-27346-w, published online 03 January 2023

The original version of this Article contained errors in the Discussion.

“Treatment-naïve POAG eyes had lower SSI than control eyes, even after controlling for bIOP, while there was no significant difference in SP A1 among control, treatment-naïve, and treated POAG eyes.”

now reads:

“Treatment-naïve POAG eyes had higher SSI than control eyes, even after controlling for bIOP, while there was no significant difference in SP A1 among control, treatment-naïve, and treated POAG eyes.”

“Our results (lower SSI) suggested that the cornea in the treatment-naïve POAG eyes was stiffer, even if the influence of IOP was excluded, further supporting the contribution of biomechanical factors other than IOP to glaucoma pathogenesis.”

now reads:

“Our results (higher SSI) suggested that the cornea in the treatment-naïve POAG eyes was stiffer, even if the influence of IOP was excluded, further supporting the contribution of biomechanical factors other than IOP to glaucoma pathogenesis.”

The original Article has been corrected.

